# Anticancer Effects of MAPK6 siRNA‐Loaded PLGA Nanoparticles in the Treatment of Breast Cancer

**DOI:** 10.1111/jcmm.70309

**Published:** 2025-01-17

**Authors:** Ceyhun Toruntay, Fatma Sayan Poyraz, Seda Susgun, Emrah Yucesan, Banu Mansuroglu

**Affiliations:** ^1^ Department of Molecular Biology and Genetics, Faculty of Science and Letters Istanbul Technical University Istanbul Turkiye; ^2^ Department of Molecular Biology and Genetics, Graduate School of Science and Engineering Yildiz Technical University Istanbul Turkiye; ^3^ Department of Molecular Biology and Genetics, Faculty of Arts and Sciences Yildiz Technical University Istanbul Turkiye; ^4^ Department of Genetics, Institute of Health Sciences Istanbul University Istanbul Turkiye; ^5^ Department of Medical Biology, Faculty of Medicine Bezmialem Vakif University Istanbul Turkiye; ^6^ Department of Neurogenetics, Institute of Neurological Sciences Istanbul University‐Cerrahpasa Istanbul Turkiye

**Keywords:** anticancer, breast cancer, MAPK6, PLGA nanoparticle, siRNA

## Abstract

siRNA‐loaded nanoparticles open new perspectives for cancer treatment. *MAPK6* is upregulated in breast cancer and is involved in cell growth, differentiation and cell cycle regulation. Herein, we aimed to investigate the anticancer effects of *MAPK6* knockdown by using MAPK6 siRNA‐loaded PLGA nanoparticles (siMAPK6‐PLGA‐NPs) in MCF‐7 breast cancer cells. After the synthesis and characterisation of nanoparticles, treatment concentrations were determined with cytotoxicity assay. Subsequently, *MAPK6* knockdown and anticancer effects of siMAPK6‐PLGA‐NPs were evaluated by in vitro assays. siMAPK6‐PLGA‐NPs have been determined to suppress *MAPK6* expression efficiently. In vitro studies revealed that siMAPK6‐PLGA‐NPs significantly reduced the migration, proliferation and colony‐forming ability and enhanced the apoptosis in MCF‐7 cells. Taken together, siMAPK6‐PLGA‐NPs exhibited robust and promising anticancer effects against MCF‐7 cells. Our findings demonstrated that siRNA‐loaded PLGA nanoparticles have great potential for breast cancer treatment and *MAPK6* gene may be the therapeutic target in breast cancer.

## 1 Introduction

Breast cancer (BC) is the most prevalent malignancy in women to receive a diagnosis and is one of the main causes of cancer‐related mortality [1]. BC is mainly divided into 4 molecular subgroups according to the presence of human epidermal growth factor receptor 2 (HER2), oestrogen receptor (ER) and progesterone receptor (PR) in the plasma membrane or cytosol. These subgroups are as follows: Luminal A, Luminal B, HER2‐positive and Triple‐negative breast cancers [2]. Luminal A is the most common subtype of BC. It consists of cancer cells that contain at least one of the ER and PR receptors and, however, do not contain HER2 receptor [2, 3]. In cancer treatment, treatment options such as surgery, chemotherapy, radiotherapy and immunotherapy are preferred alone or in combination. However, these treatment options are not applicable to all cancer types, various problems reduce the therapeutic effect (such as tumour heterogeneity, multidrug resistance etc.), and various side effects may occur during and after treatment [4, 5, 6, 7].

Mitogen‐activated protein kinase (MAPK) signalling pathways and MAPKs are highly associated with the onset and progression of cancer [8]. The MAPK/ERK signalling pathway plays a central role in regulating a wide variety of cellular processes such as cell proliferation, programmed cell death, cellular stress responses and cellular movements [9, 10, 11]. MAPK6, also known as extracellular signal‐regulated kinase 3 (ERK3), is an atypical MAPK and is involved in cell growth, cell cycle regulation and cell differentiation [12, 13]. It has been found that *MAPK6* is upregulated in BC and various other types of cancer, and knockdown of *MAPK6* inhibits the proliferation, migration and invasion of cancer cells [14, 15, 16, 17, 18].

RNA interference (RNAi) process, in which the expression of the target gene is suppressed by small non‐coding RNA molecules, is a relatively new approach for cancer therapy [19, 20]. Small interfering RNA (siRNA) suppresses the expression of target gene via RNAi with low off‐target effect [19]. However, the low cellular uptake of negatively charged siRNA, its vulnerable structure against enzymatic activity and short intracellular half‐lives are major challenges to siRNA‐based therapeutics [21, 22]. Therefore, efficient and biocompatible delivery systems are required for the therapeutic application of siRNA molecules.

Nanoparticles are among the most widely used delivery systems for systemic delivery of siRNA therapeutics [23]. Polymeric nanoparticles are frequently preferred for therapeutic agent delivery due to their ease of therapeutic agent loading, ease of synthesis, biodegradability and controlled release properties [24, 25]. Studies have shown that siRNA‐loaded polymeric nanoparticles exhibit high therapeutic efficacy [26, 27]. Among the polymers used in nanoparticle synthesis, poly(lactic‐co‐glycolic acid) (PLGA), which stands out with its high biocompatibility and is approved by the US Food and Drug Administration (FDA) and the European Medicines Agency (EMA), is one of the most favourable polymers for systemic delivery of siRNA therapeutics [28, 29].

In this study, we produced MAPK6 siRNA‐loaded PLGA nanoparticles (siMAPK6‐PLGA‐NPs) to investigate their potential anticancer effects against the MCF‐7 BC cell line. After the synthesis, we performed the characterisation of the nanoparticles and determined their suitability for usage. Herein, we revealed that siMAPK6‐PLGA‐NPs significantly decreased the migration, proliferation and colony‐forming ability and significantly increased the apoptosis in MCF‐7 cells. Taken together, we believe that *MAPK6* gene is the therapeutic target in BC and that MAPK6 siRNA‐loaded PLGA nanoparticles have high potential in BC treatment.

## 2 Materials and Methods

### 2.1 Materials

Detailed information about the materials used in the study is given in the Supporting Information.

### 2.2 Synthesis of PLGA Nanoparticles

Synthesis of siMAPK6‐PLGA‐NPs and scrambled siRNA‐loaded PLGA nanoparticles (siSCR‐PLGA‐NPs) were performed by double emulsion‐solvent evaporation (W_1_/O/W_2_) technique. Primarily, the lyophilised siRNAs were solubilised with 1 mL RNase‐free water. 30 mg PLGA was dissolved in 0.5 mL DCM. Then, 100 μL MAPK6 siRNA solution (20 nmol/mL) was added into this PLGA solution and gently mixed by pipetting. This solution forming the organic phase was added to 4 mL of 3% PVA solution with a syringe and sonicated for 90 s at 45 W power on an ice bath (Bandelin Sonopuls, Berlin, Germany). The primary emulsion (W_1_/O) was added dropwise to 35 mL of 0.1% PVA solution on the magnetic stirrer running at 800 rpm with a syringe to form the external phase. For the removal of the organic solvent, the double emulsion (W_1_/O/W_2_) was stirred for 4 h at 800 rpm. Then, resultant PLGA nanoparticle solution was centrifuged at 10000× *g* for 40 min at 4°C (Hettich Universal 32R, Tuttlingen, Germany). The pellet containing precipitated nanoparticles was washed three times with 35 mL deionised water to remove PVA and obtained supernatants were collected for characterisation analyses. PLGA nanoparticles were frozen at −80°C for 24 h and then lyophilised (Telstar Cryodos‐80, Terrasa, Spain). Lyophilised PLGA nanoparticles were stored at −20°C. Also, blank PLGA nanoparticles (Blank‐PLGA‐NPs) were synthesised by single emulsion‐solvent evaporation (O/W) technique without siRNA addition.

### 2.3 Characterisation of PLGA Nanoparticles

#### 2.3.1 Encapsulation Efficiency, Reaction Yield and Drug Loading

Encapsulation Efficiency (EE%) and Drug Loading (DL%) values were determined by using the indirect method. The *λ*_max_ values of the siRNA solutions were determined as 259 nm by UV–vis spectroscopy (Shimadzu UV‐1800, Kyoto, Japan). Then, calibration curves of the siRNAs at 259 nm were generated. Thus, the amounts of unencapsulated siRNA found in the collected supernatants were determined by UV–vis spectrophotometer (Shimadzu UV‐1800) at 259 nm. EE%, DL% and reaction yield (RY%) values were calculated according to the equations below.

(1) $$\mathrm{EE}\%=\frac{\text{Amount of siRNA loaded into the nanoparticle}\;\left(\mathrm{mg}\right)}{\text{Initial amount of siRNA}\;\left(\mathrm{mg}\right)}\times 100$$

(2) $$\mathrm{DL}\%=\frac{\text{Amount of siRNA loaded into the nanoparticle}\;\left(\mathrm{mg}\right)}{\text{Amount of lyophilized nanoparticles}\;\left(\mathrm{mg}\right)}\times 100$$

(3) $$\mathrm{RY}\%=\frac{\text{Amount of lyophilized nanoparticles}\;\left(\mathrm{mg}\right)}{\text{Initial amount of PLGA}+\text{siRNA}\left(\mathrm{mg}\right)}\times 100$$

#### 2.3.2 Mean Particle Size, Polydispersity Index and Zeta Potential

Mean particle size (Z‐Ave) and polydispersity index (PDI) values were analysed by dynamic light scattering (DLS) method using Zetasizer 4000 (Malvern Instruments, Worcestershire, UK). Zeta potential (ζ) values were analysed by phase analysis light scattering (PALS) method using Zetasizer Nano ZS (Malvern Instruments). All measurements were carried out in triplicates at 25°C ± 0.1°C.

#### 2.3.3 Nanoparticle Morphology

The morphology and size of PLGA nanoparticles were analysed and visualised by field emission scanning electron microscopy (SEM) and atomic force microscopy (AFM). For the SEM images, nanoparticles were coated with gold–palladium and imaged at 60,000 × magnification using Carl‐Zeiss EVO LS10 SEM (Jena, Germany) at 10.00 kV acceleration voltage. For AFM images, nanoparticles were analysed in dynamic mode and imaged in 1 × 1 μm area using Shimadzu SPM‐9500J3 AFM.

#### 2.3.4 Fourier Transform Infrared Spectroscopy (FTIR)

The bonds and functional groups on the surface of the PLGA nanoparticles were examined by Nicolet iS10 FTIR Spectrometer (Thermo Fisher Scientific). Infrared spectra were obtained using the attenuated total reflection (ATR) accessory with 16 scans per sample in the region of 4000 to 500 cm^−1^ with 4 cm^−1^ resolution.

#### 2.3.5 In Vitro Release

For evaluation of in vitro release, 2 mg lyophilised nanoparticles were suspended in 2 mL phosphate‐buffered saline (PBS) at physiological pH (7.4). Nanoparticle suspensions were kept in a shaker incubator at 37°C with a speed of 200 rpm during the experiment. At specific time intervals, nanoparticle suspensions were centrifuged at 10000× *g* for 20 min at 4°C. Then, 100 μL supernatants were collected. The removed supernatants were replaced with 100 μL fresh PBS. The amounts of siRNA in the supernatants were determined by UV‐vis spectrophotometer (Shimadzu UV‐1800) at 259 nm. The results were evaluated cumulatively according to the calibration curves.

### 2.4 Cell Culture

MCF‐7 breast cancer cell line was from American Type Culture Collection (ATCC HTB‐22, Maryland, USA). MCF‐7 cells were cultured in DMEM/F12 medium that supplemented with 10% FBS and 0.2% Primocin and incubated at 37°C in a 5% CO_2_ atmosphere during the study.

### 2.5 Cytotoxicity Assay

In vitro cytotoxicity of produced PLGA nanoparticles was evaluated by MTT assay. The cell viability percentage was calculated according to Equation 4. After the calculation, 40% inhibiting concentration (IC_40_) values were determined. Please refer to the Supporting Information for the detailed description of the procedure.

(4) $$\text{Cell Viability}\%=\frac{{\mathrm{OD}}_{570}\;\text{of treated cells}}{\;{\mathrm{OD}}_{570}\;\text{of untreated cells}\;\left(\text{negative control}\right)}\times 100$$

### 2.6 RNA Isolation and Quantitative Real‐Time Polymerase Chain Reaction

The relative *MAPK6* expression level in MCF‐7 cells was analysed by quantitative real‐time polymerase chain reaction (qRT‐PCR). Please refer to the Supporting Information for the detailed description of the procedure.

### 2.7 Enzyme‐Linked ImmunoSorbent Assay

The relative MAPK6 protein level in MCF‐7 cells was evaluated by commercial human enzyme‐linked immunosorbent assay (ELISA) kit (BTLab), in accordance with the manufacturer's instructions. Please refer to the Supporting Information for the complete procedure.

### 2.8 Wound Healing Assay

The effect of siMAPK6‐PLGA‐NPs treatment on the migration of MCF‐7 cells was evaluated with wound healing assay (scratch assay). The complete procedure is given in the Supporting Information.

### 2.9 Cell Proliferation Assay

The effect of siMAPK6‐PLGA‐NPs treatment on the proliferation of MCF‐7 cells was determined by immunocytochemical Proliferating cell nuclear antigen (PCNA) detection. The percentage of PCNA‐positive cells was calculated according to the Equation 5. Detailed description of the procedure is given in the Supporting Information.

(5) $$\text{PCNA positive cells}\%=\frac{\text{Number of PCNA positive cells}}{\text{Number of total cells}}\times 100$$

### 2.10 Colony Formation Assay

The effect of siMAPK6‐PLGA‐NPs treatment on the ability to form colonies of MCF‐7 cells was evaluated by colony formation assay. Please refer to the Supporting Information for the detailed description of the procedure.

### 2.11 Flow Cytometry

The effect of siMAPK6‐PLGA‐NPs treatment on the apoptosis of MCF‐7 cells was evaluated by flow cytometry using the Muse Annexin V & Dead Cell assay kit (Merck Millipore), following the manufacturer's instructions. The complete procedure is given in the Supporting Information.

### 2.12 Statistical Analysis

Analysis and plotting of all the data were done with GraphPad Prism 8.0 (California, USA). The data was shown as the mean ± SD. The Shapiro–Wilk test was used to check data normality. One‐way or two‐way analysis of variance (ANOVA) with Tukey's post hoc test was used for statistical analyses of multiple groups. *p*‐values of less than 0.05 were deemed statistically significant.

## 3 Results

### 3.1 Characterisation of PLGA Nanoparticles

In this study, siMAPK6‐PLGA‐NPs and siSCR‐PLGA‐NPs were prepared by the W_1_/O/W_2_ technique. Also, Blank‐PLGA‐NPs were prepared by the O/W technique without siRNA addition. EE% and DL% were determined by using the indirect method. EE% of siMAPK6‐PLGA‐NPs and siSCR‐PLGA‐NPs were determined as 87.75% and 81%, respectively. DL% of siMAPK6‐PLGA‐NPs and siSCR‐PLGA‐NPs were determined as 0.18% and 0.22%, respectively. Also, RY% of siMAPK6‐PLGA‐NPs, siSCR‐PLGA‐NPs and Blank‐PLGA‐NPs were calculated as 42.9%, 36.3% and 34.3%, respectively.

**FIGURE 1:**
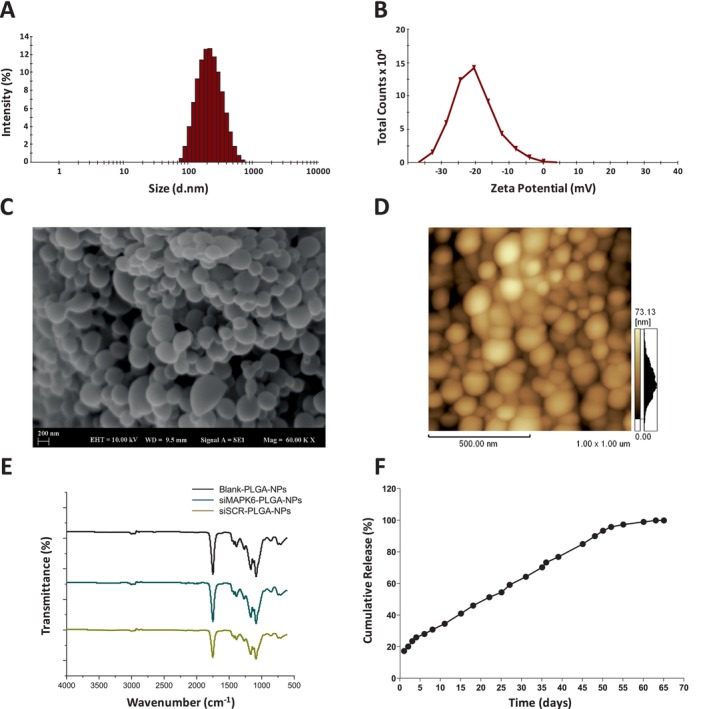
Characterisation of siMAPK6‐PLGA‐NPs. (A) Hydrodynamic size distribution of siMAPK6‐PLGA‐NPs. (B) Zeta potential of siMAPK6‐PLGA‐NPs. (C) The SEM image of siMAPK6‐PLGA‐NPs. (D) The AFM image of siMAPK6‐PLGA‐NPs. (E) FTIR spectra of Blank‐PLGA‐NPs, siMAPK6‐PLGA‐NPs and siSCR‐PLGA‐NPs. (F) In vitro release profile of siMAPK6‐PLGA‐NPs.

Subsequently, Z‐Ave, PDI and ζ of PLGA nanoparticles were measured. Z‐Ave, PDI and ζ for siMAPK6‐PLGA‐NPs were 198.6 ± 1.27 nm, 0.177 ± 0.01 and − 20.5 ± 0.46 mV (Figure 1A,B), for siSCR‐PLGA‐NPs were 192.2 ± 0.61 nm, 0.144 ± 0.02 and 23.5 ± 0.42 mV (Figure S1A,B), and for Blank‐PLGA‐NPs 154.9 ± 1.47 nm, 0.128 ± 0.02 and − 24.7 ± 2.34 mV (Figure S1C,D), respectively.

The morphology of PLGA nanoparticles was analysed and visualised by using SEM and AFM. The images of SEM and AFM revealed that siMAPK6‐PLGA‐NPs, siSCR‐PLGA‐NPs and Blank‐PLGA‐NPs have spherical and homogenous morphology, and smooth surface (Figure 1C,D and Figure S2A–D). The average size determined by using AFM for siMAPK6‐PLGA‐NPs was 73.13 nm, for siSCR‐PLGA‐NPs was 62.34 nm and for Blank‐PLGA‐NPs was 58.79 nm (Figure 1D and Figure S2C,D). The mean particle size (Z‐Ave) determined by the DLS method using Zetasizer indicates the hydrodynamic diameter of the PLGA nanoparticles. During the measurement, these nanoparticles are in a water‐containing state. However, the average size determined by AFM is obtained after the evaporation of the water. Therefore, the average sizes of PLGA nanoparticles determined by AFM are smaller.

Encapsulation success of siRNAs into PLGA nanoparticles and interactions on the surface of PLGA nanoparticles were investigated by FTIR spectroscopy. As can be seen in Figure 1E, the FTIR spectra of siMAPK6‐PLGA‐NPs, siSCR‐PLGA‐NPs and Blank‐PLGA‐NPs were compared and no difference was observed in the characteristic peaks indicating the functional groups in the structure of the PLGA copolymer. In the FTIR spectrum of siMAPK6‐PLGA‐NPs and siSCR‐PLGA‐NPs, there were characteristic peaks of PLGA, as in the spectrum of Blank‐PLGA‐NPs. These are the intense peak indicating C‐O‐C stretching between 1090 and 1100 cm^−1^, the intense peak indicating C=O stretching between 1755 and 1760 cm^−1^ and the peak indicating C‐H stretching between 2900 and 3100 cm^−1^ [30, 31].

Furthermore, the release profiles of siRNA‐loaded PLGA nanoparticles were evaluated by in vitro release study (Figure 1F and Figure S3). It was determined that 17.24% of encapsulated siRNAs from siMAPK6‐PLGA‐NPs and 16.67% from siSCR‐PLGA‐NPs were released within 24 h with the effect of burst release. The cumulative release of siRNA from siMAPK6‐PLGA‐NPs and siSCR‐PLGA‐NPs reached 30.80% and 31.36% within 7 days, respectively. Moreover, the cumulative release of siRNA from siMAPK6‐PLGA‐NPs and siSCR‐PLGA‐NPs reached 98.91% and 99.16% at 60th day, respectively.

### 3.2 Effect of siMAPK6‐PLGA‐NPs on the Viability of MCF‐7 Cells

**FIGURE 2:**
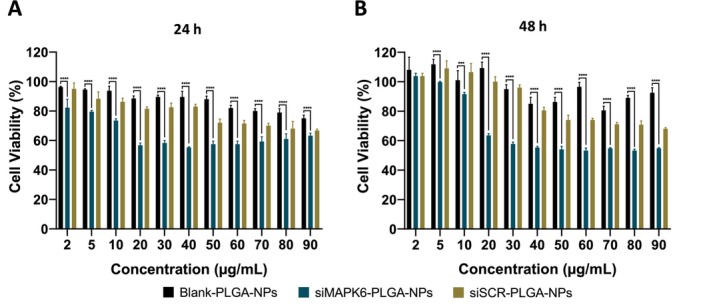
Cytotoxicity of Blank‐PLGA‐NPs, siMAPK6‐PLGA‐NPs and siSCR‐PLGA‐NPs on MCF‐7 cells after (A) 24 and (B) 48 h. Cell viabilities were determined by MTT assay after 24 and 48 h of incubation. Data are shown as mean ± SD (*n* = 4). Asterisks (***p < 0.001 and ****p < 0.0001) demonstrate the significance of the differences.

In vitro cytotoxicity of siMAPK6‐PLGA‐NPs, siSCR‐PLGA‐NPs and Blank‐PLGA‐NPs on MCF‐7 cells was evaluated by MTT assay (Figure 2A,B). Blank‐PLGA‐NPs and siSCR‐PLGA‐NPs did not exhibit high cytotoxicity. Cell viability in Blank‐PLGA‐NPs treated cells did not decrease below 75% and 77% for 24 and 48 h treatment, respectively. Similarly, cell viability in siSCR‐PLGA‐NPs treated cells did not decrease below 67% and 68% for 24 and 48 h treatment, respectively. siMAPK6‐PLGA‐NPs showed slightly higher cytotoxicity, and cell viability decreased below 60% after both 24 and 48 h treatment. Thus, the IC_40_ values were determined. The IC_40_ values of siMAPK6‐PLGA‐NPs were determined as 20 and 30 μg/mL for 24 and 48 h treatment, respectively.

### 3.3 Effect of siMAPK6‐PLGA‐NPs on MAPK6 mRNA and Protein Levels

**FIGURE 3:**
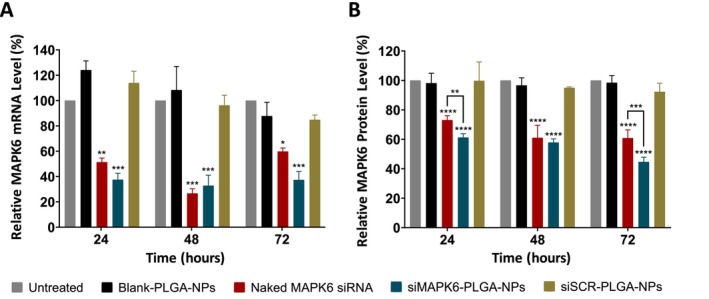
Effects of siMAPK6‐PLGA‐NPs on MAPK6 mRNA and protein levels in MCF‐7 cells. (A) Relative *MAPK6* gene expression at 24, 48 and 72 h were determined by qRT‐PCR. (B) Relative MAPK6 protein level at 24, 48 and 72 h were determined by ELISA. Data are given as the percentage average ± SD (*n* = 3), assuming the mRNA and protein levels of untreated cells as 100%. Asterisks (*p < 0.05, **p < 0.01, ***p < 0.001 and ****p < 0.0001) demonstrate the significance compared to the untreated cells.

The siRNA‐mediated knockdown of *MAPK6* with siMAPK6‐PLGA‐NPs was evaluated by qRT‐PCR (Figure 3A). There were no significant changes in *MAPK6* gene expression after 24, 48 and 72 h treatment for Blank‐PLGA‐NPs or siSCR‐PLGA‐NPs compared to the untreated. Naked MAPK6 siRNA significantly reduced *MAPK6* gene expression by 48.8% for 24 h, 73.4% for 48 h and 40.2% for 72 h compared to the untreated (*p* < 0.01, *p* < 0.001, and *p* < 0.05, respectively). Meanwhile, siMAPK6‐PLGA‐NPs significantly reduced *MAPK6* gene expression by 63.5% for 24 h, 68.7% for 48 h and 63% for 72 h compared to the untreated (*p* < 0.001). Thus, the lowest level of MAPK6 mRNA for 24 and 72 h treatment was in siMAPK6‐PLGA‐NPs treated cells. These results indicated that MAPK6 siRNA successfully suppressed the *MAPK6* gene expression.

Subsequently, the effect of this siRNA‐mediated knockdown of MAPK6 on protein level was evaluated by ELISA (Figure 3B). Similarly, there were no significant changes in MAPK6 protein levels after 24, 48 and 72 h treatment for Blank‐PLGA‐NPs or siSCR‐PLGA‐NPs compared to the untreated. Naked MAPK6 siRNA significantly reduced MAPK6 protein level by 26.9% for 24 h, 39% for 48 h and 39.1% for 72 h compared to the untreated (*p* < 0.0001). Meanwhile, siMAPK6‐PLGA‐NPs significantly reduced MAPK6 protein level by 38.7% for 24 h, 42.2% for 48 h and 55.3% for 72 h compared to the untreated (*p* < 0.0001). Moreover, siMAPK6‐PLGA‐NPs treatment for 24 and 72 h significantly reduced MAPK6 protein levels compared to the naked MAPK6 siRNA treatment (*p* < 0.01, and *p* < 0.001, respectively). These results indicated that siMAPK6‐PLGA‐NPs reduced MAPK6 expression at the mRNA and protein level.

### 3.4 Effect of siMAPK6‐PLGA‐NPs on the Migration of MCF‐7 Cells

**FIGURE 4:**
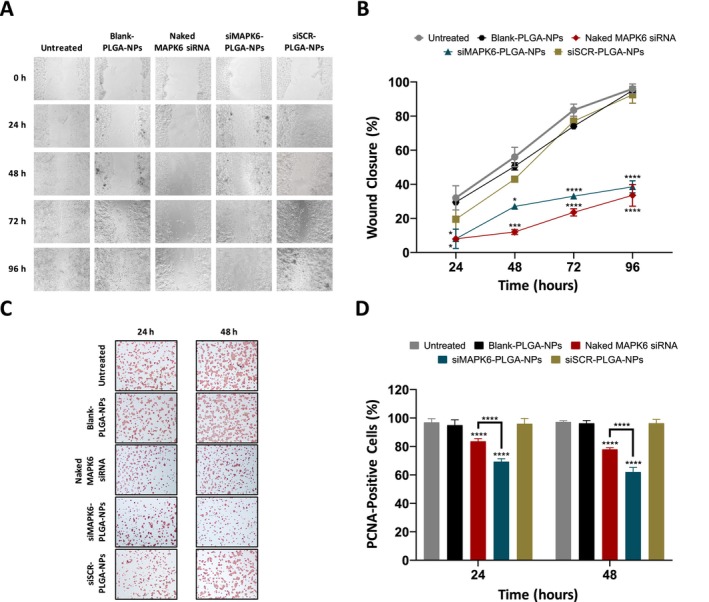
Effects of siMAPK6‐PLGA‐NPs on migration and proliferation of MCF‐7 cells. (A) Migration of MCF‐7 cells was evaluated by wound healing assay. Representative wound healing images at 0, 24, 48, 72, and 96 h at 4X magnification. (B) Percentages of wound area closure at 0, 24, 48, 72 and 96 h. (C) Proliferation of MCF‐7 cells was evaluated by immunocytochemical PCNA detection. PCNA immunocytochemical staining images of MCF‐7 cells at 24 and 48 h. (D) Quantitative representation of PCNA‐positive MCF‐7 cells PCNA as a percentage. Data are shown as mean ± SD (*n* = 3). Asterisks (*p < 0.05, ***p < 0.001 and ****p < 0.0001) demonstrate the significance of the differences.

The effect of siMAPK6‐PLGA‐NPs on the migration of MCF‐7 cells was evaluated by wound healing assay (Figure 4A,B). Blank‐PLGA‐NPs and siSCR‐PLGA‐NPs did not significantly affect the percentage of wound closure at 24, 48, 72 and 96 h compared to the untreated. As can be seen in Figure 4B, siMAPK6‐PLGA‐NPs significantly reduced the percentage of wound closure at 24, 48, 72 and 96 h compared to the untreated (*p* < 0.05, *p* < 0.05, *p* < 0.0001, and *p* < 0.0001, respectively). Similarly, naked MAPK6 siRNA significantly reduced the percentage of wound closure at 24, 48, 72 and 96 h compared to the untreated (*p* < 0.05, *p* < 0.001, *p* < 0.0001, and *p* < 0.0001, respectively). These results indicated that the knockdown of *MAPK6* by siMAPK6‐PLGA‐NPs or naked MAPK6 siRNA treatment reduced the migration of MCF‐7 cells.

### 3.5 Effect of siMAPK6‐PLGA‐NPs on the Proliferation of MCF‐7 Cells

The effect of siMAPK6‐PLGA‐NPs on the proliferation of MCF‐7 cells was evaluated by immunocytochemical PCNA detection (Figure 4C,D). Blank‐PLGA‐NPs and siSCR‐PLGA‐NPs treatment for 24 and 48 h did not significantly affect the PCNA‐positive cell percentage compared to the untreated. The PCNA‐positive cell percentages of the untreated group were 96.9% at 24 h and 97.2% at 48 h. Naked MAPK6 siRNA significantly reduced the PCNA‐positive cell percentage to 83.6% at 24 h and 78% at 48 h compared to the untreated (Figure 4D; *p* < 0.0001). Meanwhile, siMAPK6‐PLGA‐NPs significantly reduced the PCNA‐positive cell percentage to 69.3% at 24 h and 62.1% at 48 h compared to the untreated (Figure 4D; *p* < 0.0001). Importantly, a significant reduction in the percentages of PCNA‐positive cells was observed after 24 and 48 h of siMAPK6‐PLGA‐NPs treatment compared to naked MAPK6 siRNA treatment (Figure 4D; *p* < 0.0001). These results indicated that the knockdown of *MAPK6* by siMAPK6‐PLGA‐NPs significantly reduced the proliferation of MCF‐7 cells.

### 3.6 Effect of siMAPK6‐PLGA‐NPs on the Colony‐Forming Ability of MCF‐7 Cells

**FIGURE 5:**
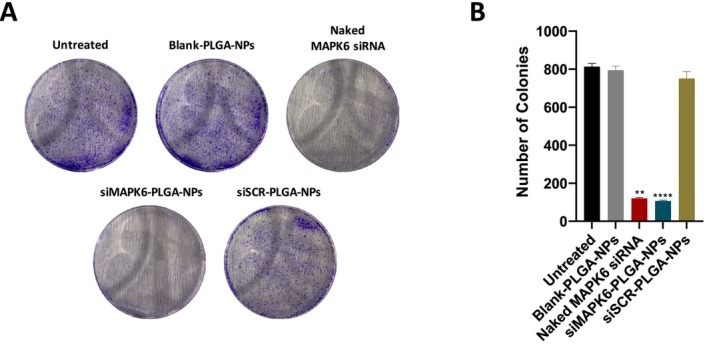
Effects of siMAPK6‐PLGA‐NPs on colony‐forming ability of MCF‐7 cells. (A) Colony‐forming ability of MCF‐7 cells was evaluated by colony formation assay. Representative images of colony formation assay. (B) Number of cell colonies per well. Data are given as the percentage average ± SD (*n* = 3). Asterisks (**p < 0.01 and ****p < 0.0001) demonstrate the significance compared to the untreated cells.

The effect of siMAPK6‐PLGA‐NPs on the ability to form colonies of MCF‐7 cells was evaluated by colony formation assay (Figure 5A). Blank‐PLGA‐NPs and siSCR‐PLGA‐NPs treatment did not significantly affect the colony formation compared to the untreated. The colony number of the untreated group was 813. Naked MAPK6 siRNA significantly reduced the number of colonies to 120 compared to the untreated. As demonstrated in Figure 5B, although naked MAPK6 siRNA treatment reduced colony formation, siMAPK6‐PLGA‐NPs treatment exerted a more significant reduction (*p* < 0.01, and *p* < 0.0001, respectively). The colony number of siMAPK6‐PLGA‐NPs treated group was 106. These results indicated that the knockdown of *MAPK6* by siMAPK6‐PLGA‐NPs significantly reduced the colony formation of MCF‐7 cells.

### 3.7 Effect of siMAPK6‐PLGA‐NPs on the Apoptosis of MCF‐7 Cells

**FIGURE 6:**
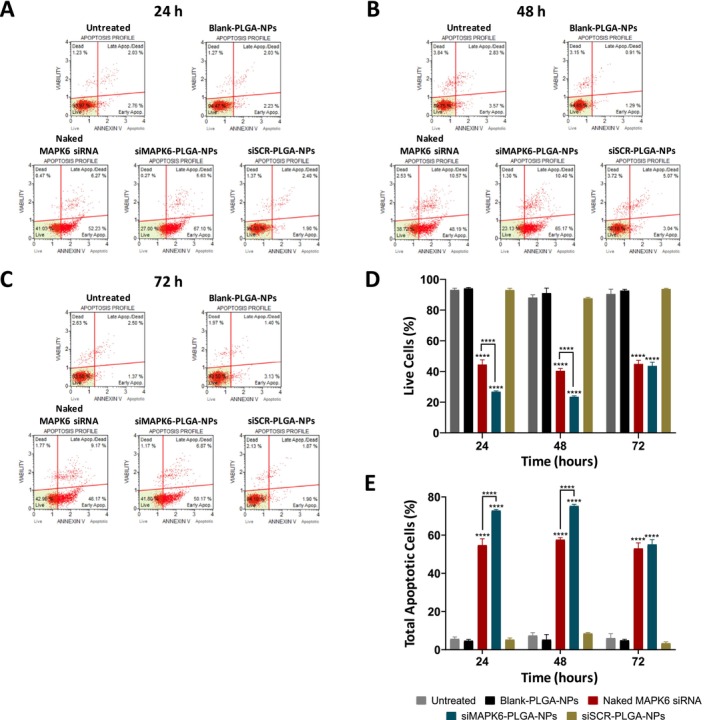
Effects of siMAPK6‐PLGA‐NPs on apoptosis of MCF‐7 cells. Apoptosis of MCF‐7 cells was evaluated by flow cytometry. Representative flow cytometry plots at (A) 24, (B) 48 and (C) 72 h after treatment. (D) Percentages of live cells at 24, 48 and 72 h. (E) Percentages of total apoptotic cells at 24, 48 and 72 h. Data are given as the percentage average ± SD (*n* = 3). Asterisks (****p < 0.0001) demonstrate the significance of the differences.

The effect of siMAPK6‐PLGA‐NPs on the apoptosis of MCF‐7 cells was evaluated by flow cytometry (Figure 6A–C). Blank‐PLGA‐NPs and siSCR‐PLGA‐NPs treatment for 24, 48 and 72 h did not significantly affect the percentage of live cells compared to the untreated. The percentages of live cells of the untreated group were 93.2% at 24 h, 88.1% at 48 h and 90.5% at 72 h. As can be seen in Figure 6D, naked MAPK6 siRNA significantly reduced the percentages of live cells to 44.5% at 24 h, 40.4% at 48 h and 44.9% at 72 h compared to the untreated (*p* < 0.0001). siMAPK6‐PLGA‐NPs significantly reduced the percentages of live cells to 26.8% at 24 h, 23.5% at 48 h and 43.6% at 72 h compared to the untreated (*p* < 0.0001). Importantly, a significant reduction in the percentages of live cells was observed after 24 and 48 h of siMAPK6‐PLGA‐NPs treatment compared to naked MAPK6 siRNA treatment (*p* < 0.0001).

Similarly, there was no significant difference in the percentages of total apoptotic cells (early apoptotic + late apoptotic) between the untreated cells and cells treated with Blank‐PLGA‐NPs and siSCR‐PLGA‐NPs (Figure 6E). The percentages of total apoptotic cells of the untreated group were 5.7% at 24 h, 7.4% at 48 h and 6.1% at 72 h. As can be seen in Figure 6E, naked MAPK6 siRNA significantly increased the percentages of total apoptotic cells to 54.8% at 24 h, 57.6% at 48 h and 53% at 72 h compared to the untreated (*p* < 0.0001). siMAPK6‐PLGA‐NPs significantly increased the percentages of total apoptotic cells to 72.8% at 24 h, 75.3% at 48 h and 55.1% at 72 h compared to the untreated (*p* < 0.0001). Importantly, a significant increase in the percentages of total apoptotic cells was observed after 24 and 48 h of siMAPK6‐PLGA‐NPs treatment compared to naked MAPK6 siRNA treatment (*p* < 0.0001). These results indicated that the knockdown of *MAPK6* by siMAPK6‐PLGA‐NPs significantly increased the apoptosis of MCF‐7 cells.

## 4 Discussion

BC is the most prevalent malignancy in women [1]. Current cancer treatments are preferred based on factors such as the type and severity of the cancer [4]. Therefore, treatment options are not applicable to all BC subgroups and these treatments may cause severe side effects and multidrug resistance [6, 32, 33]. In recent years, studies on alternative treatment strategies have gathered speed. There are recent studies indicating that flavonoids such as Ononin have an anticancer effect in Triple‐negative breast cancer [34]. Additionally, it has been determined that suppression of upregulated genes in breast cancer causes crucial anticancer effects in breast cancer [35]. At this point, RNAi offers a great alternative therapeutic approach for cancer patients where conventional treatments are inadequate [36]. siRNAs may suppress cancer‐promoting target genes via RNAi with low off‐target effect [19]. However, there are limitations in the therapeutic application of siRNAs, such as efficacy, duration of silencing effect, and nuclease stability. Thus, overcoming the limitations of siRNA‐based cancer therapy requires an efficient and biocompatible delivery system that will increase the cellular uptake of siRNAs and protect them from nuclease activity [21, 22]. PLGA nanoparticles, which stand out among the polymeric nanoparticles with their high biocompatibility, are frequently preferred in the delivery of siRNA therapeutics [22, 37, 38]. There are several studies showing that siRNA‐loaded PLGA nanoparticles successfully suppress target gene expression in cancer cells. The study by Piao et al. revealed that CRIF1 siRNA‐loaded PLGA nanoparticles suppressed *CRIF1* expression in MCF‐7 breast cancer cells, and this suppression reduced tumour growth and development [39]. Another study by Risnayanti et al. showed that MDR1 and BCL2 siRNA‐loaded PLGA nanoparticles suppressed the expression of *MDR1* and *BCL2* in ovarian cancer cells, and this suppression overcame the chemoresistance of paclitaxel and cisplatin [37]. Therefore, we preferred PLGA nanoparticles as the siRNA delivery system in this study.

Herein, we successfully generated siMAPK6‐PLGA‐NPs to deliver MAPK6 siRNA to MCF‐7 cells. We also generated siSCR‐PLGA‐NPs and Blank‐PLGA‐NPs to evaluate the therapeutic effect of siMAPK6‐PLGA‐NPs. The siMAPK6‐PLGA‐NPs had a high encapsulation efficiency of 87.75% with a reaction yield (RY%) of 42.9%. Moreover, it was determined that the MAPK6 siRNAs were successfully encapsulated by no difference in the characteristic peaks of PLGA copolymer between the FTIR spectra. The siMAPK6‐PLGA‐NPs had a hydrodynamic diameter of 198.6 ± 1.27 nm with a small PDI of 0.177 ± 0.01, which was suitable for in vivo therapeutic application (Figure 1A). Because nanoparticles, which are greater than 200 nm in size, can activate the complement system. Thus, nanoparticles can be quickly cleared [40, 41]. Also, siMAPK6‐PLGA‐NPs had a zeta potential of −20.5 ± 0.46 mV (Figure 1B). It was determined that siMAPK6‐PLGA‐NPs had spherical, smooth and homogeneous morphology by SEM and AFM images (Figure 1C,D). Besides, 17.24% of encapsulated siRNA was released from siMAPK6‐PLGA‐NPs in the neutral environment within 24 h, because of burst release (Figure 1F). The nanoparticle dose to be applied was determined with the MTT assay as in recent studies [42]. The IC_40_ values of siMAPK6‐PLGA‐NPs were 20 and 30 μg/mL for 24 and 48 h treatment, respectively (Figure 2A,B). siMAPK6‐PLGA‐NPs treatment was not able to inhibit cell viability by 50%; therefore, IC_40_ values were determined as done by Gutiérrez‐Pacheco et al. [43].

MAP K6 is an atypical MAPK and is involved in important processes for cancer such as cell growth and cell differentiation [12, 13]. It has been revealed to be upregulated in BC and various other cancers [15, 16]. Long et al. have revealed that *MAPK6* is upregulated in lung cancer and knockdown of *MAPK6* inhibited invasion [44]. Similarly, Wu et al. have demonstrated that *MAPK6* is upregulated in non‐small cell lung cancer [16]. Tan et al. have revealed that knockdown of *MAPK6* reduced the proliferation and migration of vascular smooth muscle cells [45]. The studies by Wu et al. and Huang et al. have demonstrated that knockdown of *MAPK6* inhibited the proliferation, migration, and invasion of cervical cancer cells [17, 18]. Interestingly, Lv et al. have revealed that *MAPK6* is upregulated in BC and this upregulation is associated with poor survival of BC patients [15]. Also, Bogucka et al. have found that knockdown of *MAPK6* inhibited the metastasis of BC cells [14]. In the present study, MAPK6 was identified as a therapeutic target in MCF‐7 breast cancer cells. siMAPK6‐PLGA‐NPs effectively suppressed *MAPK6* gene expression after treatment for 24, 48, and 72 h (Figure 3A). This suppression at the level of gene expression was also largely reflected in the level of MAPK6 protein. While the most effective suppression of gene expression was observed at 48 h, the lowest MAPK6 protein level was observed at 72 h (Figure 3B). This may be due to the proteins being more stable than mRNAs. Proteins generally continue to be present when the mRNA encoding them is removed [46]. Surprisingly, we determined that naked MAPK6 siRNA successfully suppressed MAPK6 expression at the mRNA and protein level, although not as much as siMAPK6‐PLGA‐NPs (Figure 3B).

Finally, we investigated the anticancer effects of siMAPK6‐PLGA‐NPs against MCF‐7 cells. The effect of the treatments on cell migration was evaluated by wound healing assay, the effect on cell proliferation by immunocytochemical PCNA detection, the effect on the ability to form colonies by colony formation assay and the effect on apoptosis by flow cytometry. We determined that siMAPK6‐PLGA‐NPs significantly reduced the migration, proliferation and ability to form colonies of MCF‐7 cells, and significantly increased apoptosis of MCF‐7 cells (Figures 4, 5, 6). Naked MAPK6 siRNA, which was determined to effectively knock down *MAPK6*, also led to similar effects. However, the siMAPK6‐PLGA‐NPs showed a stronger anticancer activity compared to naked MAPK6 siRNA except for wound healing assay. Moreover, we determined that the more effective suppression of siMAPK6‐PLGA‐NPs at 72 h compared to naked MAPK6 siRNA did not lead to a significant difference in terms of anticancer effect. This finding suggests that suppressing *MAPK6* expression after a certain level does not increase the anticancer effect. We also determined that siSCR‐PLGA‐NPs and Blank‐PLGA‐NPs had no effect on the migration, proliferation, ability to form colonies, and apoptosis of MCF‐7 cells. Taken together, these results indicated that PLGA nanoparticles were successful in the delivery of siRNA therapeutics and knockdown of the *MAPK6* with siMAPK6‐PLGA‐NPs led to significant anticancer effects against MCF‐7 BC cells.

In conclusion, MAPK6 siRNA‐loaded PLGA nanoparticles were produced for the delivery of MAPK6 siRNAs into MCF‐7 BC cells. It was determined that the produced siMAPK6‐PLGA‐NPs successfully knockdown *MAPK6* expression in MCF‐7 cells. Subsequently, in vitro analyses showed that siMAPK6‐PLGA‐NPs significantly reduced the migration, proliferation and ability to form colonies of MCF‐7 cells, and significantly increased their apoptosis. Therefore, siMAPK6‐PLGA‐NPs exhibited effective and promising anticancer effects against MCF‐7 cells. Furthermore, these findings strongly support the idea that *MAPK6* gene may be a potential therapeutic target in BC.

## Author Contributions

**Ceyhun Toruntay:** conceptualization, data curation, formal analysis, investigation, methodology, visualization, writing – original draft. **Fatma Sayan Poyraz:** conceptualization, data curation, formal analysis, investigation, methodology. **Seda Susgun:** formal analysis, methodology. **Emrah Yucesan:** methodology, resources, validation. **Banu Mansuroglu:** conceptualization, data curation, funding acquisition, project administration, resources, supervision, writing – review and editing.

## Ethics Statement

*Approval of the research protocol by an Institutional Reviewer Board*: N/A. *Informed consent*: N/A. *Registry and the registration no. of the study/trial*: N/A. *Animal studies*: N/A.

## Conflicts of Interest

The authors declare no conflicts of interest.

## Supporting information


Data S1.

## Data Availability

The data of the study are available upon request.
