# Supplementary material for: Anticancer Effects of MAPK6 siRNA‐Loaded PLGA Nanoparticles in the Treatment of Breast Cancer
Source: J Cell Mol Med. 2025 Jan 17;29(2):e70309. doi: 10.1111/jcmm.70309 (PMC11740982; doi:10.1111/jcmm.70309)
Supplement: Supplementary file 1 — Data S1. [file JCMM-29-e70309-s001.docx]

**Anticancer effects of MAPK6 siRNA-loaded PLGA nanoparticles in the treatment of breast cancer**

Ceyhun Toruntay^a,b,*^, Fatma Sayan Poyraz^c^, Seda Susgun^d,e^, Emrah Yucesan^f^, Banu Mansuroglu^c^

^a^ *Istanbul Technical University, Faculty of Science and Letters, Department of Molecular Biology and Genetics, Istanbul, Turkiye*

^b^ *Yildiz Technical University, Graduate School of Science and Engineering, Department of Molecular Biology and Genetics, Istanbul, Turkiye*

^c^ *Yildiz Technical University, Faculty of Arts and Sciences, Department of Molecular Biology and Genetics, Istanbul, Turkiye*

^d^ *Istanbul University, Institute of Health Sciences, Department of Genetics, Istanbul, Turkiye*

^e^ *Bezmialem Vakif University, Faculty of Medicine, Department of Medical Biology, Istanbul, Turkiye*

^f^ *Istanbul University-Cerrahpasa, Institute of Neurological Sciences, Department of Neurogenetics, Istanbul, Turkiye*

* Corresponding author: Ceyhun Toruntay

*E-mail address*: [ctoruntay@itu.edu.tr](mailto:ctoruntay@itu.edu.tr) / [ceyhuntoruntay@gmail.com](mailto:ctoruntay@itu.edu.tr)

**Extended Materials and Methods**

**Materials**

PLGA (lactide:glycolide = 50:50, molecular weight = 24-38 kDa), polyvinyl alcohol (PVA) and dichloromethane (DCM) were purchased from Sigma–Aldrich (Poole, UK). MAPK6 siRNA (5’-UACUGAUGUUGUUGAUAAATT-3’, 5’-UUUAUCAACAACAUCAGUAGG-3’) and scrambled negative control siRNA (5’-UUCUCCGAACGUGUCACGU[dT][dT]-3’, 5’-ACGUGACACGUUCGGAGAA[dT][dT]-3’) were purchased from Qiagen (Hilden, Germany). Dulbecco's Modified Eagle Medium/Nutrient Mixture F-12 (DMEM/F-12), fetal Bovine Serum (FBS), Trypsin-EDTA (0.25%), Dulbecco's phosphate-buffered saline (DPBS), 3-(4,5-Dimethylthiazol-2-yl)-2,5-Diphenyltetrazolium Bromide (MTT) and Dimethyl sulfoxide (DMSO) were purchased from Gibco/Thermo Fisher Scientific (Massachusetts, USA). Primocin was purchased from InvivoGen (California, USA). The Monarch Total RNA Miniprep kit, ProtoScript First Strand cDNA Synthesis kit and Luna Universal qPCR Master Mix were purchased from New England Biolabs (Massachusetts, USA). Primers for MAPK6 (Forward: 5’-TTTGCTGAAATGCTGACTGG-3’; Reverse: 5’-ATCCATGGGGCTAAATGTCA-3’) and GAPDH (Forward: 5’-CTGGGCTACACTGAGCACC-3’; Reverse: 5’-AAGTGGTCGTTGAGGGCAATG-3’) were purchased from Sentromer DNA Technologies (Istanbul, Turkiye). Human MAPK6 ELISA kit was purchased from BTLab (Shanghai, China). Purified anti-human/mouse/rat PCNA antibody was purchased from BioLegend (California, USA). The Histostain-Plus kit, AEC Substrate kit and Histomount Mounting solution were purchased from Invitrogen/Thermo Fisher Scientific (Massachusetts, USA). The Mayer′s Hematoxylin solution and paraformaldehyde were purchased from Sigma–Aldrich (Poole, UK). Crystal violet was purchased from Merck Millipore (Darmstadt, Germany). The Muse Annexin V & Dead Cell Assay Kit was purchased from Luminex (Texas, USA).

**Cytotoxicity Assay**

At a density of 1 × 10^4^ cells per well, MCF-7 cells were seeded into 96-well plates and incubated for 24 h. After the incubation, the medium was changed with a fresh medium that contained 2-90 μg/mL siMAPK6-PLGA-NP, siSCR-PLGA-NP and Blank-PLGA-NP, and cells were incubated for 24 and 48 h. Also, certain wells were left untreated as negative controls. Following the 24 and 48 h of incubation, 10 μL MTT (5 mg/mL) was added to each well and plates were incubated for 3 h at 37°C with 5% CO_2_. The supernatants were removed. 100 μl DMSO was added to the wells and plates were incubated for 15 min at 37°C to solubilize the formazan crystals. Then, absorbance was read at 570 nm by using a MultiSkan GO Microplate Reader (Thermo Fisher Scientific, Massachusetts, USA). The experiment was carried out in quadruplicate, and data were presented as mean and standard deviations (SD) of quadruplicates. The percentage of cell viability was calculated according to Equation S1. After the calculation, 40% inhibiting concentration (IC_40_) values were determined.

$$Cell Viability \%=\frac{{OD}_{570} of treated cells}{{OD}_{570} of untreated cells (negative control)}\times100 (S1)$$

**RNA Isolation and Quantitative Real-Time Polymerase Chain Reaction**

At a density of 3 × 10^5^ cells per well, cells were seeded into 6-well plates and incubated for 24 h at 37°C with 5% CO_2_. Then, the medium was changed with a fresh medium that contained IC_40_ concentration of siMAPK6-PLGA-NPs, siSCR-PLGA-NPs, Blank-PLGA-NPs and naked MAPK6 siRNA. Certain wells were left untreated as negative controls. Cells were incubated for 24, 48 and 72 h at 37°C, 5% CO_2_. After the incubation, cells were washed twice with DPBS. After washing, cells were scraped with cell scrapers. Total RNA was purified from MCF-7 cells by using the Monarch Total RNA Miniprep kit (New England Biolabs) in accordance with the manufacturer's protocol. RNA purity and quantity were detected by MultiSkan GO Microplate Reader (Thermo Fisher Scientific). RNA was transcribed into complementary DNA (cDNA) by using ProtoScript First Strand cDNA Synthesis kit (New England Biolabs). After the cDNA synthesis, Quantitative Real-Time Polymerase Chain Reaction (qRT-PCR) was performed on a CFX96 Touch Real-Time PCR Detection System (Bio-Rad Laboratories, California, USA) by using Luna Universal qPCR Master Mix (New England Biolabs) according to the manufacturer's protocol. *GAPDH* was used as a housekeeping gene. The primers used were as follows: MAPK6 forward: 5’-TTTGCTGAAATGCTGACTGG-3’ and reverse: 5’-ATCCATGGGGCTAAATGTCA-3’; GAPDH forward: 5’-CTGGGCTACACTGAGCACC-3’ and reverse: 5’- AAGTGGTCGTTGAGGGCAATG-3’. The qRT-PCR conditions were as follows: Initial denaturation 95°C for 60 s, and 40 cycles of 95°C for 15 s, 60°C for 30s. Analysis of Ct values was performed by the delta-delta Ct (2^–ΔΔCt^) method. The experiment was carried out in triplicate. Results were given as the percentage of gene expression, assuming the gene expression of untreated (control) cells as 100%.

**Enzyme-Linked ImmunoSorbent Assay**

At a density of 3 × 10^5^ cells per well, MCF-7 cells were seeded into 6-well plates and incubated for 24 h at 37°C with 5% CO_2_. Then, the medium was changed with a fresh medium that contained IC_40_ concentration of siMAPK6-PLGA-NPs, siSCR-PLGA-NPs, Blank-PLGA-NPs and naked MAPK6 siRNA. Certain wells were left untreated as negative controls. Cells were incubated for 24, 48 and 72 h at 37°C, 5% CO_2_. After the incubation, cells were washed twice with DPBS. After washing, cells were scraped with cell scrapers. Since MAPK6 is an intracellular protein, lysis of cells suspended in DPBS was performed by sonication. Then, the samples were centrifuged at 12000 x g for 15 min at 4°C to get rid of cell debris. The relative MAPK6 protein level in the samples was determined by using the human MAPK6 Enzyme-Linked ImmunoSorbent Assay (ELISA) kit (BTLab) according to the manufacturer's protocol. Absorbance at 450 nm was read by using a MultiSkan GO Microplate Reader (Thermo Fisher Scientific). The experiment was carried out in triplicate. Results were given as the percentage of protein level, assuming the protein level of untreated (control) cells as 100%.

**Wound Healing Assay**

At a density of 5 × 10^4^ cells per well, cells were seeded into 24-well plates and incubated at 37°C with 5% CO_2_ until they reached 70% confluency. Then, the medium was changed with a fresh medium that contained IC_40_ concentration of siMAPK6-PLGA-NPs, siSCR-PLGA-NPs, Blank-PLGA-NPs and naked MAPK6 siRNA. Certain wells were left untreated as negative controls. After the treatment, cell layers in the wells were scratched with a sterile 1000 µL pipette tip. Plates were incubated at 37°C with 5% CO_2_ during the experiment. The images of the wounds (scratches) were collected at the beginning (0 h) and every 24 h for 96 h under Primovert inverted cell culture microscope with an AxioCam ERc5s camera (Carl Zeiss, New York, USA). The experiment was carried out in triplicate. Wound areas were calculated using the ImageJ software. Data were shown as a percentage of wound closure.

**Cell Proliferation Assay**

At a density of 1 × 10^4^ cells per well, MCF-7 cells were seeded on coverslips in 12-well plates and incubated at 37°C with 5% CO_2_ until they reached confluency. Then, the medium was changed with a fresh medium that contained 24 and 48 h IC_40_ concentration of siMAPK6-PLGA-NPs, siSCR-PLGA-NPs, Blank-PLGA-NPs and naked MAPK6 siRNA. Certain wells were left untreated as negative controls. Cells were incubated for 24 and 48 h at 37°C, 5% CO_2_. After the incubation, cells were washed with PBS and fixed with ice-cold methanol for 5 min at −20°C. Then, serum blocking solution (Thermo Fisher Scientific) was applied for 20 min at room temperature. Blocking solution was removed and PCNA primary antibody (BioLegend) was applied at 1:100 dilution for 3 h at room temperature. Then, cells were washed with PBS. After washing, biotinylated secondary antibody and streptavidin-HRP (Thermo Fisher Scientific) were applied respectively for 20 min at room temperature. Subsequently, cells were washed with PBS and immunoreactive cells were stained with AEC Substrate kit (Thermo Fisher Scientific) for 10 min at room temperature. Then, cells were washed with distilled water and counterstaining was performed using Mayer’s hematoxylin (Sigma–Aldrich) for 15 s. Cells were washed with distilled water until the coverslips became clear. Then, coverslips were mounted onto the glass slides with a mounting solution (Thermo Fisher Scientific). Cells were examined by using a Nikon Eclipse i5 light microscope and imaged by Nikon DS-Fi1c camera (Nikon Instruments, Tokyo, Japan). 10 different areas were selected and stained/unstained cells were counted in each area. The experiment was carried out in triplicate. The percentage of PCNA-positive cells was calculated according to the following equation:

$$PCNA positive cells \%=\frac{Number of PCNA positive cells}{Number of total cells}\times100 (S2)$$

**Colony Formation Assay**

At a density of 5 × 10^2^ cells per well, MCF-7 cells were seeded into 6-well plates and incubated for 24 h at 37°C with 5% CO_2_. Then, the medium was changed with a fresh medium that contained IC_40_ concentration of siMAPK6-PLGA-NPs, siSCR-PLGA-NPs, Blank-PLGA-NPs and naked MAPK6 siRNA. Certain wells were left untreated as negative controls. Plates were incubated at 37°C, 5% CO_2_ during the experiment. After 8 days of incubation, visible colonies were formed. Cells were washed with PBS and fixed with PFA for 30 min at 4°C. Then, cells stained with 0.5% crystal violet for 30 min at room temperature. After staining, the wells were washed with distilled water and dried. Lastly, colonies were counted and imaged. Cell clusters containing 50 or more cells were defined as colonies. The experiment was carried out in triplicate.

**Flow Cytometry**

At a density of 5 × 10^4^ cells per well, MCF-7 cells were seeded into 24-well plates and incubated for 24 h at 37°C with 5% CO_2_. Then, the medium was changed with a fresh medium that contained IC_40_ concentration of siMAPK6-PLGA-NPs, siSCR-PLGA-NPs, Blank-PLGA-NPs and naked MAPK6 siRNA. Certain wells were left untreated as negative controls. Cells were incubated for 24, 48 and 72 h at 37°C, 5% CO_2_. After the incubation, cells were washed with DPBS and harvested with trypsinization. Harvested cells were centrifuged at 2000 x g for 5 min at 4°C and supernatants were removed. Then, cells were suspended with PBS, centrifuged again at 2000 x g for 5 min and resuspended with PBS. Cells were incubated with Muse Annexin V & Dead Cell reagent (Merck Millipore) according to the manufacturer's protocol. After the incubation, the percentage of healthy, apoptotic and dead cells were determined by using Muse® Cell Analyzer (Merck Millipore). The experiment was carried out in triplicate.

**
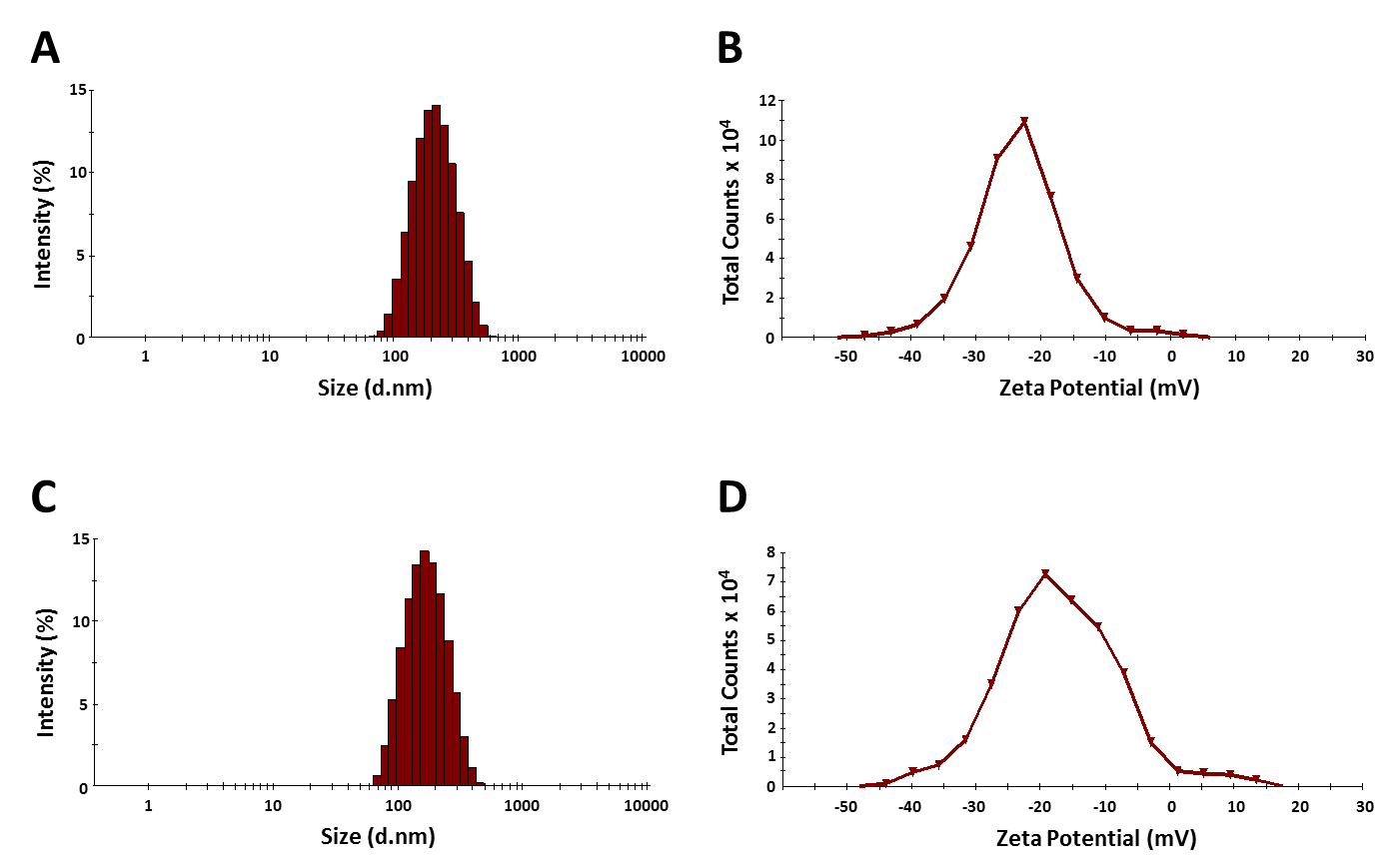
**

**Supplementary Figure S1.** Size and zeta potential of siSCR-PLGA-NPs and Blank-PLGA-NPs. (**A**) Hydrodynamic size distribution of siSCR-PLGA-NPs. (**B**) Zeta potential of siSCR-PLGA-NPs. (**C**) Hydrodynamic size distribution of Blank-PLGA-NPs. (**D**) Zeta potential of Blank-PLGA-NPs.

**
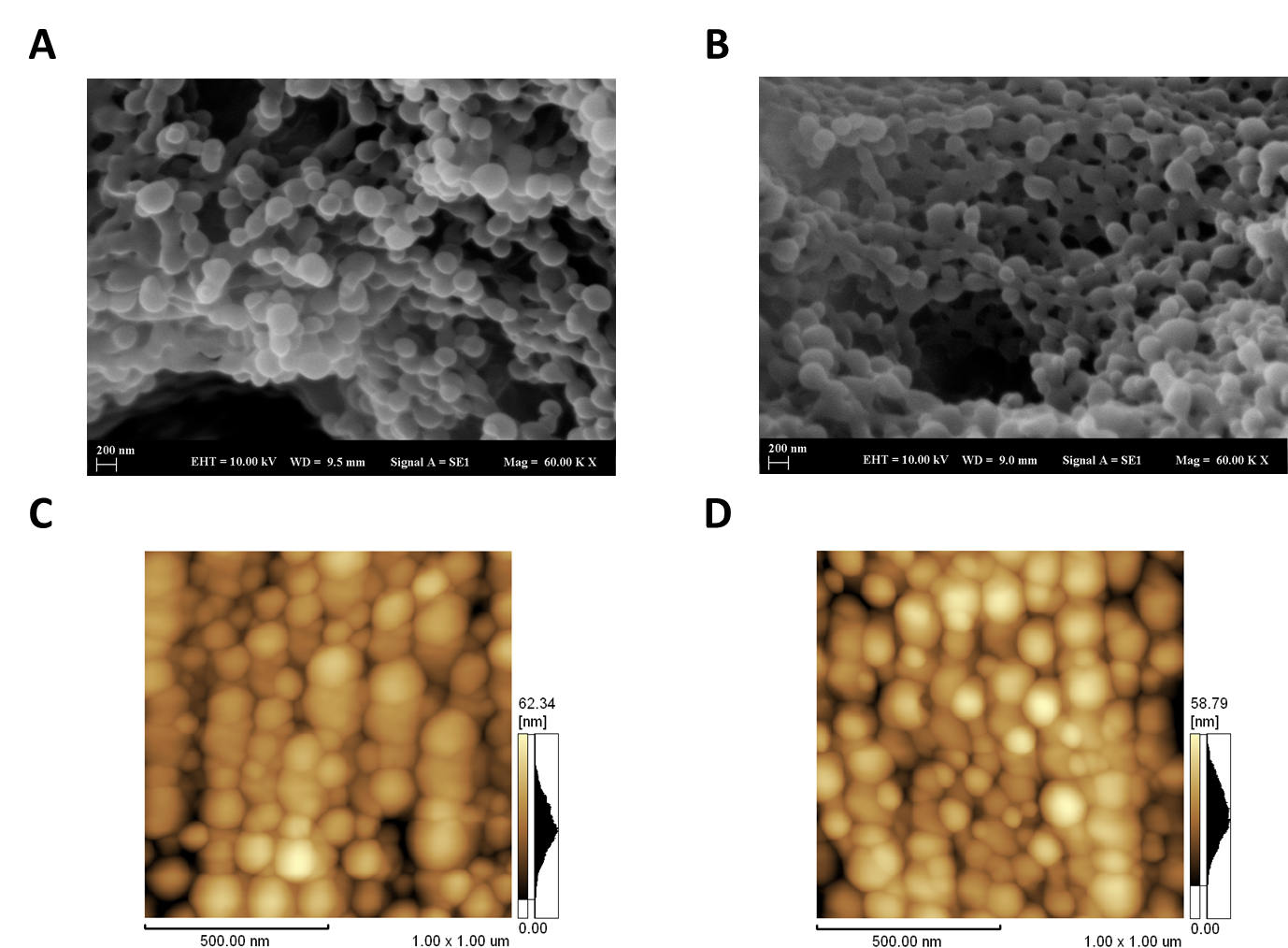
**

**Supplementary Figure S2.** Morphology of siSCR-PLGA-NPs and Blank-PLGA-NPs. (**A**) The SEM image of siSCR-PLGA-NPs. (**B**) The SEM image of Blank-PLGA-NPs. (**C**) The AFM image of siSCR-PLGA-NPs. (**D**) The AFM image of Blank-PLGA-NPs.

**Supplementary Figure S3.** *In vitro* release profile of siSCR-PLGA-NPs.
